# Developing and validating the Nursing Cultural Competence Scale in Taiwan

**DOI:** 10.1371/journal.pone.0220944

**Published:** 2019-08-13

**Authors:** Mei Hsiang Lin, Te Hsin Chang, Yu Hsia Lee, Pao Yu Wang, Li Hui Lin, Hsiu Chin Hsu

**Affiliations:** 1 Department of Nursing, National Taipei University of Nursing and Health Sciences, Taiwan, R.O.C; 2 MacKay Memorial Hospital, Taiwan, R.O.C; 3 Graduate Institute of Gerontology and Health Care Management, Chang Gung University of Science and Technology, Taiwan, R.O.C; Guangzhou University, CHINA

## Abstract

**Background:**

Culture influences personal health habits and behavior, and healthcare personnel possess different views of cultural perspectives. Currently, an appropriate instrument to assess cultural competence in clinical practice is limited. The present study aimed to develop and examine the psychometric properties of the Nursing Cultural Competence Scale (NCCS) for clinical nurses.

**Methods:**

Developing and assessing the scale was carried out in two phases: Phase I involved a qualitative research to explore the themes of nurses’ cultural competence and instrument development; Phase II established construct validity of the scale using a sample of 246 nurses in Taiwan. Data from the questionnaire were analyzed using exploratory factor analysis, confirmatory factor analysis, internal consistency and test-retest reliability. Analysis results were used to determine the reliability and validity of the developed scale.

**Results:**

The results showed four factors including cultural awareness ability, cultural action ability, cultural resources application ability, and self-learning cultural ability were generated by exploratory factor analysis, and these factors explained 62.0% of total variance. Cronbach’s α of the Nursing Cultural Competence Scale was .88, and test-retest reliability correlation was .70.

**Conclusions:**

The establishment of the tool will facilitate accurate monitoring of the cultural competence among nurses and nursing managers, which can inform the construction of nursing policies aimed at pledge cultural competence expansion.

## Introduction

Today, international migration is a global and complex phenomenon. The culturally competent healthcare structure may be augmented [1–2]. Cultural competence consists of two subconcepts, culture and competence [3]. Culture is a collection of shared characteristics that encompasses learned patterns of thought, communications, beliefs, institutions of racial, religious as well as social groups and behavior, including language, action, and ethnic or religious institutions [4– 6]. Competence implies an ongoing process that involves accepting and respecting differences and not letting one’s personal cultural beliefs have an undue influence on those whose worldview is different from one’s own [5]. Nursing cultural competence has generally been understood in a nursing capacity to promote the health and wellness of clients whose cultural backgrounds are different from that of the nurses [7].

Many researchers have reported that nursing cultural competencies can ensure that nurses would provide culturally specific information, as well as certain explicit services to clients with different cultural conflicts [3, 8]. Notably, cultural competence is considered to be a core competency as evident in the professional codes and standards of practice internationally [9]. There are more than 45 types of self-administered tools for cultural competence among the health professionals that are used in both a clinical and curricular content [10]. To the best of our knowledge, no scale has been developed with uniqueness and specificity to detect the localized experience of nursing cultural competence in Taiwan. Therefore, an urgent need for localized tools of cultural competence for nurses has emerged to assess their abilities of diversity from a cultural perspective.

A number of researchers have reported that culture could affect personal health habits and behavior, along with the cognition of, and seeking a response to healthy behavior [11–13]. Accordingly, Nielsen et al. [14], conducted a qualitative study which showed that the issue of death is often considered a taboo when discussed in public for the Chinese. From this point of view, because nurses are the contemporary healthcare providers, and place a significant role in performing cultural assessments, they must be sensitive to the delivery of culturally appropriate care [13, 15]. Young and Guo [6] stated that communication styles, cultural differences, explanatory styles, and interpreter services are several perspectives that require when in providing care for diversified populations. Additionally, as part of a cultural assessment, determining the specific values, beliefs, attitudes, and health needs of each patient are crucial [15]. For this reason, the cultural competence measurement tool requires to be recognized and transcend this different exposure to cultural diversity [16–17]. Also, the challenges of cultural diversity revolve around linguistic differences, verbal/nonverbal communication, and multigenerational differences [6]. However, the current measuring cultural competence tools focus on the personal attributes of medical care providers, without considering the cultural diversity of cases or their health outcomes [18]. Ethnic/ethnic health differences have been well described in numerous literature. Betancourt [19] pointed out that minority members are particularly affected by specific illnesses such as cardiovascular disease, diabetes, asthma, and cancer. Hence, in determining the relevance of cultural competence to health and well-being, it is noted that cultural competence in healthcare merged, in part, as a means of addressing racial and ethnic inequalities that may lead to health disparities [20].

The measurement of cultural competence includes both the general and specific cultural information, and serves as an assessment reference for health care providers [5]. Culture-specific tools assess the ability of the healthcare professionals to care for the patients’ needs from a particular cultural background [18], such as in the Cultural Self-Efficacy Scale (CSES) [21]. Whereas culture-general tools do not distinguish between cultural groups, as in the Inventory for Assessing the Process of Cultural Competence among Healthcare Professional-revised (IAPCC-R) [2]. Nevertheless, since few related instruments have demonstrated an acceptable reliability and/or validity, these assessment tools still exhibit limitations [22]. Lin et al. [23] conducted a systematical review which reviewed the English-language articles published from 1983 to 2013, as the findings showed that the psychometric properties of several instruments are regularly used to assess the cultural competence of the healthcare providers, and discovered that most of the Chinese versions of the cultural competence instruments are mostly based on previous literature or directly translated from the Western instruments. At this point, Cai et al. [22] indicated that it was necessary to develop a psychometrically sound instrument with a reasonable format and wording to present a complete picture of cultural competence in the Chinese nursing context that is distinct from those of Western countries. Along with this line, the ‘Nurses’ Multicultural Caring Competence Scale (NMCCS)’ is generally accepted as a valid measurement to investigate the cultural competence of the nursing staffs in Taiwan [24], however, it was still translated from the Western instrument. Moreover, the ‘Cultural Competence Inventory for Nurses in China (CCINC)’ [22] which contains five dimensions with 29 items has been developed for the perception of the Chinese nurses regarding cultural competence. The psychometric properties of the CCINC had showed good reliability and validity by examining Cronbach’s alpha and exploratory factor analysis. It is worth noting that as globalization continues to diversify populations, while current views of cultural competence for nurses in Taiwan have been chiefly adopted from Western cultures. The various ethnic groups have diverse medical care needs, for instance, immigrant patients who are waiting for an operation would like to bring a temple amulet with them, or request caregivers to perform religious rites in their wards because of their illnesses.

Culture is highly specific and individual, as nurses cannot design treatment plans on the basis of uniform standards when facing culturally-diverse patients. Therefore, a more suitable cultural competencies scale is needed to assess the nurses’ cultural competence and their interaction with the culturally and/or ethnically diverse patients. The aims of this study were to develop and examine the psychometric properties of the Nursing Cultural Competence Scale (NCCS) among nurses in Taiwan.

## Methods

### Design

Two phases were conducted to develop the NCCS for assessing the cultural competence of the clinical nurses. In-depth interviews were employed in Phase I to establish a large pool of potential items which were constructed as the preliminary scale, and then tested the instrument using the item analysis before defining the final relevant items of the scale. A cross-sectional with a descriptive study design was used for evaluating the psychometric properties of the final scale in Phase II.

### Setting and sample

Convenience sampling was employed to recruit participants from a variety of hospital units, including internal medicine, surgical, pediatric, intensive care units, and gynecology units. Besides that, in Taiwan, the clinical ladder system is one with a hierarchical structure that can be classified in four clinical ladder levels associated with an individual’s clinical abilities and proficiency growth. The four levels were N0/ N1 (responsible for basic nursing), N2 (critical care nursing), N3 (in charge of education and holistic nursing), and N4 (responsible for research and specialized nursing) [25]. The inclusion criteria were those registered nurses who have been employed for more than one year and are willing to share cultural experience during clinical care.

Nurses who were diagnosed with severe depression or other major illnesses (i.e., malignancies) were excluded from this study. There were 250 participants who met the inclusion criteria. Four participants who did not complete the questionnaire were excluded from this study. Therefore, a total of 246 participants completed this study (Phase II), and the response rate was 98.4%.

### Procedures

#### Initial item pool and item analysis (Phase I)

The NCCS items were derived mainly from information elicited during the 30 in-depth interviews. The years of nursing experiences in clinics varied from two to 20 years. The numbers of participants working in the general wards, emergency room, intensive care unit, and as case managers were five(17%), nine (30%), six (20%), seven (23%) and three (10%), respectively. A semi-structured interview guide was developed to explore the interviewees’ opinions on their cultural competence experiences among the clinical nurses. The average time for interviewing ranged from 30 to 70 minutes. There were four interview guidance contents including: ‘*What are the differences between the diverse health/sickness cultures*?’, ‘*What is your understanding of Taiwan’s health/sickness culture*?’, ‘*Please describe the content of some of the multicultural care your current workplace provides*.’, and ‘*In your opinion*, *how does one demonstrate the ability to provide multicultural care*?’ (S1 and S2 Files). Interview data were continuously collected until the data saturation was reached, and then no new information was recorded. Initially, 23 items were generated and categorized under five subheadings including (a) seven items addressing ‘*embarrassment when encountering different cultures*’; (b) seven items addressing ‘*awareness of value differences*’; (c) three items addressing ‘*difficulty implementing nursing work*’; (d) three items addressing ‘*seeking resources*’ and (e) three items addressing ‘*encompassing and acceptances*’.

A seven-member panel of experts, including four nursing professors with expertise in spirituality, cultural and research, and three nurses that had more than 10 years of experiences caring for foreign patients, were invited to verify the content validity of the NCCS. Content experts were asked to rate the clarity and relevance of each item using a 4-point rating scale. A score of ‘1’ indicated not adequate, while a score of ‘4’ represented very adequate. Finally, the item-level content validity index (CVI) was .99, and the scale-level CVI was .91, indicating that the scale had a very good validity. Nevertheless, based on the experts’ suggestions, four items were removed because of irrelevancy, redundancy and some wording ambiguities. This final instrument contained 19 item with a 5-point Likert response scale (i.e. 1 = ‘Rarely’, 2 = ‘occasionally’, 3 = ‘neutrally’, 4 = ‘often’, and 5 = ‘always’). Item analysis including maximizing the internal consistency and evaluating how well the items fit together to represent the potential construct of interest was conducted [26]. The corrected item- total correlation coefficients between the items ranged from .50–.71 and the correlation matrix was above .65 among the resultant 19-item panel-modified version of the NCCS. These results indicated that the items were consistent with the connotations that the overall scale was intended to measure. No items were removed, and the psychometric properties of the refined scale were then evaluated, after which a further analysis was then conducted.

#### Psychometric properties evaluation (Phase II)

A total of 246 participants completed the new NCCS (S3 File) and the Chinese version of the Nurses’ Multicultural Caring Competence Scale (NMCCS) (S4 File) in Phase II. The psychometric properties were performed on all the psychological constructs to assess their validity and reliability. The sample size for validating a scale should be based on the subject to item ratio of 5–10:1 [27–28]. All of the enrolled nurses were asked to complete the questionnaires and then place it into a box that was located outside their ward within seven days. See S5 File for the NCCS-minimal underlying data set.

### Ethical considerations

An approval was obtained from the Ethical Committee of the study hospital (approval No. 17MMHIS031e). The study purpose was fully explained to all the participants and written consent was acquired from them all, and they were also assured that the data collected was on an anonymous basis. Additionally, the participants were informed that they were not obliged to participate in the study and could withdraw at any time. The data were collected between August 2015 and July 2016.

### Instrument

The Chinese version of the NMCCS was devised by Liang et al. [24], with a widespread usage among Taiwan’s healthcare workers. The 29 item NMCCS scale with a 5-point Likert-type scoring system rated the responses from 0, representing ‘strongly disagree’ to 4, representing ‘strongly agree’. A higher score indicated a higher degree of cultural competence. This scale divided the cultural competence into four aspects, with a total of 29 items to measure the ‘cultural awareness ability’ (seven items), ‘cultural knowledge’ (eight items), ‘cultural sensitivity’ (three items), and ‘cultural skills’ (11 items). The Cronbach’s alpha was .91, showing the comprehensive psychometric properties [24]. In this study, a Cronbach’s alpha of the NMCCSC was .94 indicating a satisfactory internal consistency. The NMCCS was adopted in the present study as criteria for determining the concurrent validity of the NCCS.

### Statistical analysis

Data was analyzed using the IBM SPSS software statistical version 20.0 and the IBM SPSS Amos version 22.0 for Windows (IBM Corp., Armonk, New York, USA). The characteristics of the participants in this study were analyzed using descriptive statistics. An exploratory factor analysis (EFA) was performed to determine the construct validity of NCCS. Hair et al. [29] postulated that the factor extraction criteria used were (a) a factor loading of .50 or above, (b) an eigenvalue greater than one for each component, and (c) a minimum of three items for each factor. After the factors were rotated, the criteria of factor interpretability and factor usefulness were used to determine the number of factors [30]. The principal components analysis and varimax rotation were conducted to extracted common factor. In point of fact, the criterion-related validity of the NCCS was analyzed by using Pearson’s correlation to compute between the NCCS and the NMCCS to examine the concurrent validity of the NCCS. A confirmatory factor analysis (CFA) was performed to validate the factor structure that was constructed in the EFA. The Goodness of fit the model was evaluated using a variety of indices that were required to meet the following criteria: a χ^2^/ df ratio of lower than 3; a goodness-of-fit index (GFI), non-normed fit index (NNFI), and comparative fit index (CFI) that were all higher than .90; a root mean square error of approximation (RMSEA) and standardized root mean squared residual (SRMR) that were less than .08 [31–32]. Moreover, internal consistency was assessed using Cronbach’s alpha coefficients. Test-retest reliability was estimated using Pearson’s correlation coefficient.

## Results

There were 246 participants that completed the required questionnaire and assessment scales. Table 1 shows the participants’ demographic data. Most were female (*n* = 243, 98.8%) with a mean age of 32.30 ± 9.63 years (range, 20–58 years). The participants had been working as nurses for an average of 9.96 ± 9.52 years (range, 1–35 years). A total of 222 participants (90.2%) had experienced caring for foreign nationals, and 121 participants (49.2%) possessed a clinical ladder system that was N1.

**Table 1:** Characteristics of participant and demographics.

Variable	N	%
Gender		
Male	3	1.2
Female	243	98.8
Marital Status		
Unmarried	73	29.7
Married	173	70.3
Department		
Internal medicine	70	28.5
Surgery	50	20.3
Obstetrics & Gynecology	40	16.3
Pediatric	37	15.0
Emergency Room	49	19.9
Intensive care unit	70	28.5
Experience in caring for foreigners		
no	24	9.8
yes	222	90.2
Education		
Diploma	78	31.7
Bachelor’s degree	158	64.2
Master’s degree	10	4.1
Clinical Ladder System		
N0	18	7.3
N1	121	49.2
N2	60	24.4
N3	39	15.9
N4	8	3.3

### Construct validity

The internal structure of the 19-item NCCS was first tested using the EFA with a varimax rotation. The Kaiser-Meyer-Olkin (KMO) measure of sampling sufficiently produced a coefficient of .85, and the Bartlett’s test of sphericity reached the statistical significance (chi-square = 2147.88, *p* < .001) that were significant without violation in both the factor analysis and sampling requirements. Four factors emerged from the factor analysis that accounted for 62.0% of the explained variance of the NCCS. Factor 1 was loaded by the seven items, and named as the ‘*cultural awareness ability*’. Factor 2 consisted of six items, and named as the ‘*cultural action ability*’. Factor 3 represented the ‘*cultural resources application ability*’ and included three items. Factor 4 consisted of three items, and named as ‘*self-learning cultural ability*’. The four factors explained the variances ranging from 12.6 to 19.1% (Table 2).

**Table 2:** Factor analysis, item-total scale correlations and reliability for the NCCS.

	Items	Mean	SD	CITC	CR	Factor 1	Factor 2	Factor 3	Factor4
1.	I know clinically, individual cases or patients will reject treatment due to folk taboo	1.57	1.04	.53	13.22	**.71**	.10	.17	-.07
2.	I know clinically, individual cases or patients will mind homophonic	1.68	1.17	.54	16.69	**.68**	.08	.12	.06
3.	I know clinically, individual cases or patients will effect treatment due to special cultural events	1.71	1.21	.64	24.53	**.77**	.20	.08	.10
4.	I know clinically, individual cases or patients will think that the perineum is dirty	1.33	1.16	.51	11.98	**.54**	.31	-.01	-.01
5.	I know clinically, individual cases or patients will believe folk treatment is better than medical treatment	1.33	.93	.50	13.03	**.75**	.07	-.05	.07
6.	I know clinically, individual cases or patients will think of death as a taboo topic	2.17	1.06	.53	11.81	**.66**	.16	-.07	.16
7.	I know clinically, individual cases or patients will cause conflict in treatment due to different beliefs	1.59	1.10	.61	15.08	**.73**	.08	.21	.10
8.	When taking care of a case, I can handle misunderstandings due to language barrier	2.11	.95	.50	13.86	.23	**.59**	.13	-.05
9.	When taking care of a case, I can handle the difficulty when building nurse-patient relationship	2.34	.88	.59	14.51	.16	**.78**	.08	.11
10.	When taking care of a case, I can handle spending more time communicating	2.36	.87	.55	16.25	.17	**.81**	-.06	.12
11.	When taking care of a case, I can handle using different degrees of treatment guidelines due to cultural differences	2.07	.86	.71	14.12	.28	**.66**	.28	.22
12.	When taking care of a case, I can handle the degree of fear in individual cases or patients	2.50	.80	.53	10.91	.03	**.62**	.06	.40
13.	When taking care of a case, I can handle different levels of nursing care due to differences in patients’ religious rituals or living habits	2.09	.85	.58	13.18	.06	**.65**	.30	.21
14.	When taking care of patients of a different culture, I will look for help from social workers, religious personnel or colleagues	2.63	.94	.56	17.62	.07	.19	.21	**.84**
15.	When taking care of patients of a different culture, I will look for assistance from helpers or foreign workers	2.61	.97	.53	16.89	.11	.17	.09	**.84**
16.	When taking care of patients of a different culture, I will look for internet resources such as mobile phone applications or computer translation	2.52	1.05	.58	17.08	.12	.16	.38	**.69**
17.	When taking care of patients of a different culture, I will read books or watch medical television series for self-learning	2.24	1.03	.55	17.84	.02	.16	**.73**	.37
18.	When taking care of patients of a different culture, I will take part in language education courses that include everyday expressions or medical terms	1.76	1.10	.59	25.12	.12	.15	**.89**	.15
19.	When taking care of patients of a different culture, I will take part in cultural educational courses, such as; cultural background or diet preference or religious means	1.54	1.09	.57	22.38	.14	.13	**.87**	.12
	Eigen values					3.62	3.19	2.57	2.39
	Percentage of variance					19.07	16.81	13.51	12.60
	Cumulative of total variance explained (%)					19.07	35.88	49.40	62.00
	Cronbach’s α					.84	.83	.86	.82
	Cronbach’s α for the overall scale	.88							
	^#^Coefficient of stability (r)					.53	.55	.59	.61
	^#^Coefficient of stability (r) for the overall scale	.70							

Note. CITC: corrected item-total correlation.

CR: critical ratio.

SD: standard deviation.

NCCS: Nursing Cultural Competence Scale.

^#^n = 60.

**Fig 1:**
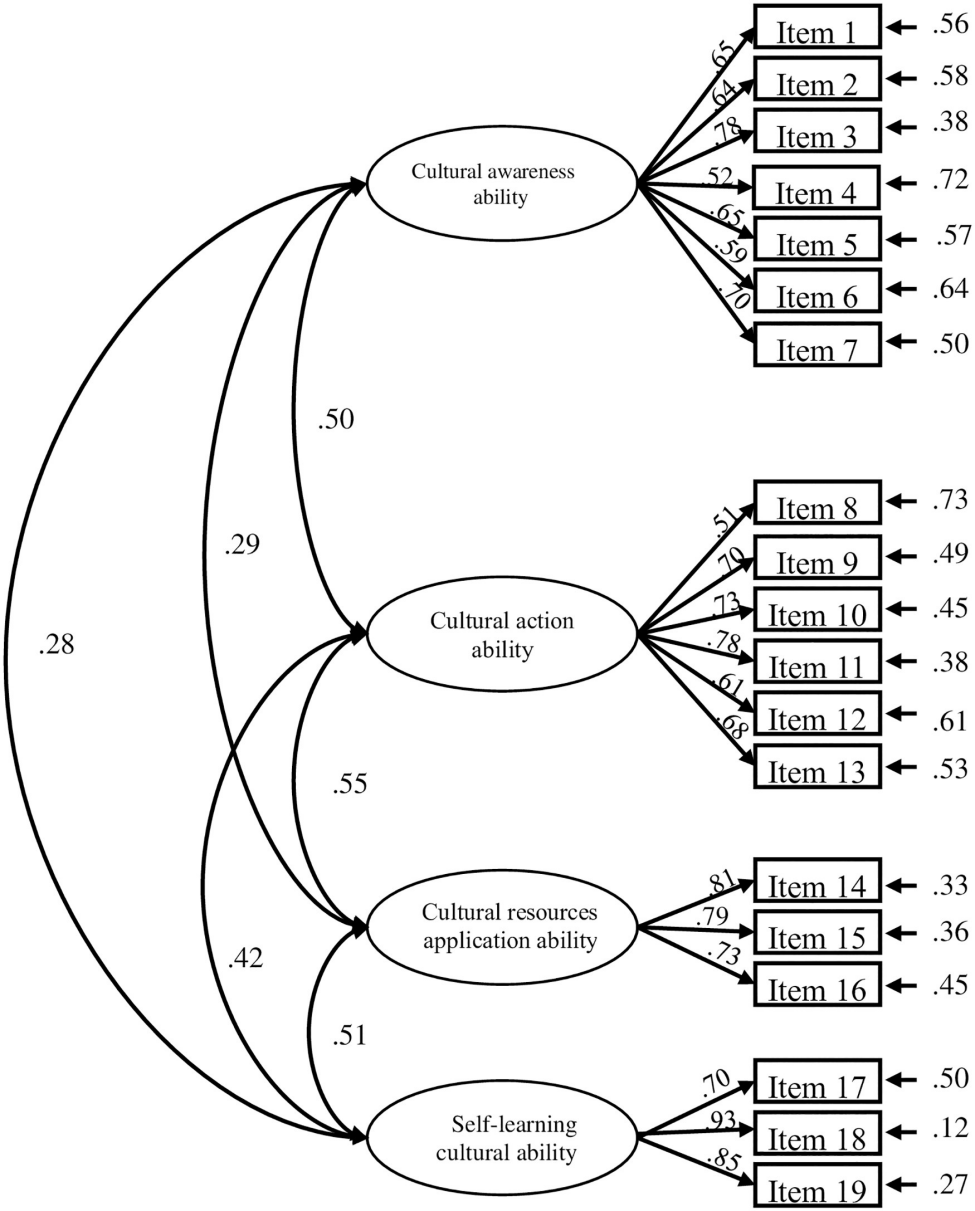
Confirmatory factor analysis of the Nursing Cultural Competence Scale.

The CFA was used to validate the factor structure that was constructed in the EFA. The model fit indices for the initial CFA were generally acceptable (i.e., χ^2^ /df = 2.50, CFI = .89, SRMR = .06, and RMSEA = .07). Convergent validity is illustrated in Table 3 and Fig 1, all standardized factor loadings exceeded the threshold of .50, and the average variance extracted (AVE) for each construct ranged from .43 to .69. Furthermore, the construct reliability (CR) for all of the constructs was greater than .82, which provided evidence for the convergent reliability of this instrument. Table 4 shows the discriminate validity of the CCNS. All of the square roots of the AVE for each construct (values in the diagonal elements) were greater than the corresponding inter-construct correlations (values below the diagonal). This suggested that the results supported the discriminate validity of the current instrument.

**Table 3:** Factor loading, and convergent validity of confirmatory factor analysis (N = 246).

Latent variables	Observational variables	λLoading	CR	AVE
Cultural awareness ability	Item 1	.65	.83	.43
	Item 2	.64		
	Item 3	.78		
	Item 4	.52		
	Item 5	.65		
	Item 6	.59		
	Item 7	.70		
Cultural action ability	Item 8	.51	.83	.46
	Item 9	.70		
	Item 10	.73		
	Item 11	.78		
	Item 12	.61		
	Item 13	.68		
Cultural resources application ability	Item 14	.81	.82	.61
	Item 15	.79		
	Item 16	.73		
Self-learning cultural ability	Item 17	.70	.87	.69
	Item 18	.93		
	Item 19	.85		

Note. All factor loadings were statistically significant at the p < .001 level.

CR = construct reliability.

AVE = average variance extraction.

λ = standardized factor loading

**Table 4:** Discriminate validity among the latent variables of confirmatory factor analysis (N = 246).

Construct	A	B	C	D
A. Cultural awareness ability	(.65)			
B. Cultural action ability	.51***	(.68)		
C. Cultural resources application ability	.29***	.55***	(.78)	
D. Self-learning cultural ability	.28***	.42***	.51***	(.83)

Note. The value in the diagonal element is the square root of AVE of each construct

***p < .001.

In Table 5, the results showed that the ‘*cultural awareness ability*’, ‘*cultural action ability*’, ‘*cultural resources application ability*’ and the ‘*self-learning cultural ability*’ correlated significantly and positively with the NMCCS *(r* = .22, .39, .33, and .44, respectively; *p* < .001).

**Table 5:** Correlations between four factors, total scores of the NCCS, and NMCCS.

Factors	NMCCS
Cultural awareness ability	.22***
Cultural action ability	.39***
Cultural resources application ability	.44***
Self- learning cultural ability	.33***
^#^NCCS	.44***

Note.

***p < .001

^#^NCCS: total scores of the NCCS.

NMCCS: total scores of the Nurses’ Multicultural Caring Competence Scale.

### Reliability

The results of this study revealed that there were good internal consistency reliability for the NCCS and it subscales. The Cronbach’s alpha coefficients for the global NCCS were .88, and the Cronbach’s alpha coefficients for the subscales were as follows: .84 for the ‘*cultural awareness ability*’, .83 for the ‘*cultural action ability*’, .86 for the ‘*cultural resources application ability*’ and .82 for the ‘*self-learning cultural ability*’, indicating a good internal consistency among the items. The stability of the NCCS was verified by fulfilling test-retest. The correlation coefficients from .53 to .61 were found in the four subscales (Table 2).

## Discussion

This newly developed scale is a culturally specific instrument designed to measure the nurses’ ability for providing various aspects of the cultural contexts of clients in Taiwan. As aforementioned there must be at least three items in one factor since too few questions would not be able to test the characteristics of the factor [30]. The newly developed NCCS has more than three items in each factor. Additionally, the four factors indicated that the total variance of the scale was 62%, which explains that the ratio is higher than the error ratio. This result shows that all four factors are representative, and hence, the newly designed cultural competence scale has a good construct validity, indicating that the NCCS can be used to evaluate the nurses’ cultural competence.

Normally, the EFA is the first step in building a scale or a new metric system [33]. The measure of sampling adequacy value was .85 using the Kaiser-Meyer-Olkin measure for the EFA, which is considered “meritorious” by Kaiser [34]. In addition, the Bartlett’s test of sphericity reached a statistical significance of (*p* < .001). In the final scale, all 19 items with factor loading > .50. According to Hair et al. [29], the factor loading of more than .50 should be approved by the criterion of selection for the scale items in order to construct a scale to analyze the results. Therefore, the NCCS met the assumptions for the factor analysis. The NCCS could be considered as a new measuring tool for Taiwan’s clinical nurses to assess their cultural competences. Furthermore, the results showed a Cronbach’s alpha was .84 for factor 1, .83 for factor 2, .86 for factor 3, .82 for factor 4 and .88 for the overall scale. DeVellis [35] pointed out that a good reliability of the scale should be above .80, and the new NCCS possessed the reliability between .82 and .86, which is thought to be more than acceptable in consistent reliability.

Four factors have emerged from the factor analysis. The first, which entitled the ‘*Cultural awareness ability*’, has a total of seven items and implies that the nurses’ self-cultural perspective allows them to care for the patients from different cultures, as well as understanding when the patients will not accept treatment due to traditional taboos, such as death, which is a subject that cannot be discussed. Traditional taboos have brought challenges to the health care system in Taiwan, and thus reduce effective communication with culturally diverse clients. The finding was congruent with previous studies which showed that taboos and other daily rituals, which restrict certain activities or mandate certain behaviors, could either harm or benefit human health and/or livelihoods [36]. From a Chinese medical perspective, the Chinese culture puts a tremendous influence on the perceptions of Taiwan’s nurses about their role as healthcare providers. Chew et al. [13] studied the impact of Chinese cultural health beliefs among 50 Malaysian Chinese from the general public of a suburban population. Those authors found that healthcare providers needed to be aware of existing beliefs and practices among the Chinese patients regarding traditional Chinese medicine. Simply stated cultural awareness signifies the ability to realize in a meaningful manner that one’s own cultural viewpoints are different from those of others, allowing one to know more about any personal understanding and bias towards foreign cultures [24].

The second factor, which entitled the ‘*Cultural action ability*’, consists of six items and points out that nurses are able to provide nursing services according to the needs of the patients’ cultural background. Nursing care involves emphasizing the practical aspects of communication and problem solving. After evaluating the information related to the cultural background for each patient, the nurses are able to deal with any misunderstandings with the patients due to the language barrier, and then provide different degrees of nursing care owing to the differences in religious rituals or lifestyles. The finding of this study was consistent with those of previous studies that addressed the health professionals abandoning their personal biases, thereby making them more able to carry out the cultural assessments and utilize [37–38].

The third factor, which entitled the ‘*Cultural resources application ability*’, has a total of three items and indicates that nurses are able to search different resources to satisfy the cultural background needs of different patients. This includes being able to find help from different professional personnel, such as religious personnel, careers, or foreign workers, and Internet resources. This finding was similar to a study that claimed nurses were reported to elicit the assistance of colleagues, patients’ caregivers, or even help from another client when dealing with various cultural aspects in clinical settings [39].

The fourth factor entitled the ‘*Self-learning cultural ability*’ has a total of three items, which indicate that nurses are able to use different methods, including reading books or taking part in cultural or language education classes, in order to enrich their understanding of different cultural information. The finding was similar to a survey conducted by Cicolini et al. [40], which showed that nurses acquired a certain level of cultural awareness and sensitivity through the experience of cross-cultural nursing in service education. However, findings of the present study were contradictory with a study by Almutairi et al. [41], which reported that some nursing staff tended to be reluctant to learn about the patients’ culture and presenting passive learning about cultural competence.

The strength of this study is that the initial items were developed using in-depth interviews with nurses in Taiwan. The qualitative research method of this study highlights the differences in the clinical cultural backgrounds between these nurses and those in the West. Cultural diversity can also refer to having different cultures respect each other’s differences [42]. In Taiwan, various groups have diverse medical care needs, including new immigrants, elderly individuals accompanied by foreign caregivers, and preoperative patients who wish to bring a temple amulet with them or wish caregivers to perform religious rites in the patients’ hospital rooms because of their illnesses. Situations such as these in which members of different groups require culturally diverse health care are extremely common in clinical settings. Therefore, results from the current study indicated that the NCCS possessed a substantial reliability and validity for assessing the cultural competence of the clinical nurses.

Another strength of this study is that multiple methods were used to establish the validity of the NCCS, including using the EFA and CFA to construct validity, and criterion-related validity. CFA is a statistical technique that is used to verify the factor structure of a set of observed variables [43]. The present study used CFA to check as to whether this new structure was acceptable with our sample. The findings showed that the model fit of the NCCS, as evaluated using GFIs, SRMR, and RMSEA was acceptable, and the CFA yielded four factors. The NCCS and its subscales were found to have good internal consistency. On the other hand, Cai et al. [22] conducted a study in which the background was familiar with this current study, Cai did not perform the CFA to validate the factor structure of the new scale.

There are several limitations of the study. Firstly, since convenience samplings and homogenous groups were employed in the present study, the generalization to the total population of clinical nurses cannot be assumed. Therefore, further testing of the NCCS is needed to cross validation on other Taiwanese clinical nurses. Secondly, 38% of the variance value was unable to be explained due to the NCCS being a new instrument with only preliminary testing, and there is still a need of ensuring additional tests to corroborate the findings of the present study.

## Conclusion

The developed NCCS has an acceptable reliability and validity when measuring the nurses’ cultural competence. Hence, this scale not only provides the nurses with an effective analysis of their clinical care cultural competence ability, but can also be used as a reference for their clinical in-service education design and planning. Furthermore, using the NCCS for assessment may provide the clinical nurses, nursing managers, and nursing educators with information about the aspects of cultural competences to guide the interventions, thereby supporting their continuous professional development.

## Supporting information

S1 File Interview guide *(English version)*. (PDF)

S2 File Interview guide *(original Chinese version)*. (PDF)

S3 File Nursing Cultural Competence Scale (NCCS) *(English version)*. (PDF)

S4 File 護理人員多元文化能力量表(NMCCS) [Nurses’ Multicultural Caring Competence Scale (NMCCS)] (*original Chinese version*). (PDF)

S5 File NCCS-minimal underlying data set. (XLSX)
