# Supplementary material for: Developing and validating the Nursing Cultural Competence Scale in Taiwan
Source: PLoS One. 2019 Aug 13;14(8):e0220944. doi: 10.1371/journal.pone.0220944 (PMC6692013; doi:10.1371/journal.pone.0220944)
Supplement: S1 File — (PDF) [file pone.0220944.s001.pdf]

S1 File. Interview guide (English version)

■What are the differences between the diverse health/sickness cultures?

■What is your understanding of Taiwan's health/sickness culture?

■Please describe the content of some of the multicultural care your current workplace provides.

■In your opinion, how does one demonstrate the ability to provide multicultural care?
