# Supplementary material for: Developing and validating the Nursing Cultural Competence Scale in Taiwan
Source: PLoS One. 2019 Aug 13;14(8):e0220944. doi: 10.1371/journal.pone.0220944 (PMC6692013; doi:10.1371/journal.pone.0220944)
Supplement: S2 File — (PDF) [file pone.0220944.s002.pdf]

S2 File.訪談指引(Chinese version)

■您的經驗中不同群體健康/疾病文化(如信念、認知、習慣、行為、反應…)的差異為何(是甚麼)?

■您了解的台灣健康/疾病文化 (如信念、認知、習慣、行為、反應…)為何?

■請您描述目前服務機構提供哪些多元文化照護(內容)?

■您認為如何做，可以滿足多元文化照護之需求?
