# Supplementary material for: Developing and validating the Nursing Cultural Competence Scale in Taiwan
Source: PLoS One. 2019 Aug 13;14(8):e0220944. doi: 10.1371/journal.pone.0220944 (PMC6692013; doi:10.1371/journal.pone.0220944)
Supplement: S3 File — (PDF) [file pone.0220944.s003.pdf]

S3 File. Nursing Cultural Competence Scale (NCCS)

1.	I know clinically, individual cases or patients will reject treatment due to folk taboo
2.	I know clinically, individual cases or patients will mind homophonic
3.	I know clinically, individual cases or patients will effect treatment due to special cultural events
4.	I know clinically, individual cases or patients will think that the perineum is dirty
5.	I know clinically, individual cases or patients will believe folk treatment is better than medical treatment
6.	I know clinically, individual cases or patients will think of death as a taboo topic
7.	I know clinically, individual cases or patients will cause conflict in treatment due to different beliefs
8.	When taking care of a case, I can handle misunderstandings due to language barrier
9.	When taking care of a case, I can handle the difficulty when building nurse-patient relationship
10.	When taking care of a case, I can handle spending more time communicating
11.	When taking care of a case, I can handle using different degrees of treatment guidelines due to cultural differences
12.	When taking care of a case, I can handle the degree of fear in individual cases or patients
13.	When taking care of a case, I can handle different levels of nursing care due to differences in patients' religious rituals or living habits
14.	When taking care of patients of a different culture, I will look for help from social workers, religious personnel or colleagues
15.	When taking care of patients of a different culture, I will look for assistance from helpers or foreign workers

16.	When taking care of patients of a different culture, I will look for internet resources such as mobile phone applications or computer translation
17.	When taking care of patients of a different culture, I will read books or watch medical television series for self-learning
18.	When taking care of patients of a different culture, I will take part in language education courses that include everyday expressions or medical terms
19.	When taking care of patients of a different culture, I will take part in cultural educational courses, such as; cultural background or diet preference or religious means
