# Supplementary material for: Developing and validating the Nursing Cultural Competence Scale in Taiwan
Source: PLoS One. 2019 Aug 13;14(8):e0220944. doi: 10.1371/journal.pone.0220944 (PMC6692013; doi:10.1371/journal.pone.0220944)
Supplement: S4 File — (PDF) [file pone.0220944.s004.pdf]

S4 File

護理人員多元文化照護能力量表

| 題號 | 內 容 | 非常同意 | 同意 | 普通 | 不同意 | 非常不同意 |
|---|---|---|---|---|---|---|
| 1 | 一個人的信念或行為會受到其文化背景的影響 | ☐ | ☐ | ☐ | ☐ | ☐ |
| 2 | 來自不同文化背景的人，其價值觀往往有差異 | ☐ | ☐ | ☐ | ☐ | ☐ |
| 3 | 多數人的健康/疾病信念或行為會受到文化背景的影響 | ☐ | ☐ | ☐ | ☐ | ☐ |
| 4 | 了解個案的文化背景對護理照護是重要的 | ☐ | ☐ | ☐ | ☐ | ☐ |
| 5 | 在了解不同文化時，不同個人的接受度有差異 | ☐ | ☐ | ☐ | ☐ | ☐ |
| 6 | 了解個案的文化背景，可以增進護理照護品質 | ☐ | ☐ | ☐ | ☐ | ☐ |
| 7 | 護理人員對健康/疾病的認知受到護理教育的影響 | ☐ | ☐ | ☐ | ☐ | ☐ |
| 8 | 我能說出不同族群間特有的健康問題 | ☐ | ☐ | ☐ | ☐ | ☐ |
| 9 | 我能舉例說明與不同文化背景個案溝通的技巧 | ☐ | ☐ | ☐ | ☐ | ☐ |
| 10 | 我了解不同文化群體對其健康信念/行為之詮釋 | ☐ | ☐ | ☐ | ☐ | ☐ |
| 11 | 我能收集到不同文化關於健康/疾病的知識和信息 | ☐ | ☐ | ☐ | ☐ | ☐ |
| 12 | 我熟悉與健康/疾病相關的文化知識或理論 | ☐ | ☐ | ☐ | ☐ | ☐ |
| 13 | 我能解釋個案健康/疾病的信念或行為與文化間可能的相關性 | ☐ | ☐ | ☐ | ☐ | ☐ |
| 14 | 我能比較不同文化背景個案的健康/疾病的信念 | ☐ | ☐ | ☐ | ☐ | ☐ |
| 15 | 我能了解不同文化背景個案的照護需求 | ☐ | ☐ | ☐ | ☐ | ☐ |
| 16 | 我會尊重不同族群文化間的差異 | ☐ | ☐ | ☐ | ☐ | ☐ |
| 17 | 不論我的個案採取哪一種健康照護方式，我認為皆有其優點 | ☐ | ☐ | ☐ | ☐ | ☐ |
| 18 | 我能接受不同文化群體關於健康/疾病的信念或行為 | ☐ | ☐ | ☐ | ☐ | ☐ |
| 19 | 我能運用溝通技巧於不同文化背景的個案 | ☐ | ☐ | ☐ | ☐ | ☐ |
| 20 | 我能了解不同文化背景個案的非語言表達 | ☐ | ☐ | ☐ | ☐ | ☐ |
| 21 | 執行護理活動前，我會完整地蒐集與個案相關的文化背景資料 | ☐ | ☐ | ☐ | ☐ | ☐ |
| 22 | 對我而言，蒐集不同文化背景個案的健康/疾病信念或行為資料是容易的 | ☐ | ☐ | ☐ | ☐ | ☐ |
| 23 | 我能解釋文化對個案的健康/疾病信念或行為的影響 | ☐ | ☐ | ☐ | ☐ | ☐ |
| 24 | 我能向不同族群的個案說明影響其健康/疾病信念或行為的文化因素 | ☐ | ☐ | ☐ | ☐ | ☐ |
| 25 | 我能依個案文化背景來建立護理措施 | ☐ | ☐ | ☐ | ☐ | ☐ |
| 26 | 執行護理活動時，我能滿足不同文化背景個案的需求 | ☐ | ☐ | ☐ | ☐ | ☐ |
| 27 | 我能教導其他護理同仁健康/疾病相關的文化知識 | ☐ | ☐ | ☐ | ☐ | ☐ |
| 28 | 我能教導其他護理同仁與不同文化背景個案溝通的技巧 | ☐ | ☐ | ☐ | ☐ | ☐ |
| 29 | 我能教導其他護理同仁為不同文化背景個案擬定照護計畫 | ☐ | ☐ | ☐ | ☐ | ☐ |
